# Persistence of Environmental DNA in Freshwater Ecosystems

**DOI:** 10.1371/journal.pone.0023398

**Published:** 2011-08-08

**Authors:** Tony Dejean, Alice Valentini, Antoine Duparc, Stéphanie Pellier-Cuit, François Pompanon, Pierre Taberlet, Claude Miaud

**Affiliations:** 1 SPYGEN, Savoie Technolac - BP 274, Le Bourget-du-Lac, France; 2 Laboratoire d'Ecologie Alpine, UMR CNRS 5553, Université de Savoie, Le Bourget-du-Lac, France; 3 Parc Naturel Régional Périgord-Limousin, La Coquille, France; 4 Laboratoire d'Ecologie Alpine, UMR CNRS 5553, Université Grenoble I, Grenoble, France; Argonne National Laboratory, United States of America

## Abstract

The precise knowledge of species distribution is a key step in conservation biology. However, species detection can be extremely difficult in many environments, specific life stages and in populations at very low density. The aim of this study was to improve the knowledge on DNA persistence in water in order to confirm the presence of the focus species in freshwater ecosystems. Aquatic vertebrates (fish: Siberian sturgeon and amphibian: Bullfrog tadpoles) were used as target species. In control conditions (tanks) and in the field (ponds), the DNA detectability decreases with time after the removal of the species source of DNA. DNA was detectable for less than one month in both conditions. The density of individuals also influences the dynamics of DNA detectability in water samples. The dynamics of detectability reflects the persistence of DNA fragments in freshwater ecosystems. The short time persistence of detectable amounts of DNA opens perspectives in conservation biology, by allowing access to the presence or absence of species e.g. rare, secretive, potentially invasive, or at low density. This knowledge of DNA persistence will greatly influence planning of biodiversity inventories and biosecurity surveys.

## Introduction

The precise knowledge of species distribution is a key point for conservation strategies, especially when the focal species are invasive, threatened or endangered [1]–[4]. However, its detection may be extremely difficult in many environments, at specific life stages and in populations at very low densities [5], [6]. To overcome this problem, DNA barcoding was recently used in order to detect species through extracellular DNA present in environmental samples, coming from cell lysis or living organism excretion or secretion [7]. This method allows species presence detection, without any contact (e.g. visual, auditory) when the only available indicators are hair, faeces or urine left behind by the organisms. For example, faeces and hair samples were used for monitoring the recent wolf range expansion in France and Switzerland [8]. In aquatic ecosystems, Ficetola et al. [5] proposed a new methodology for species detection using environmental DNA from freshwater samples. The aim was to detect the American bullfrog (*Rana catesbeiana* = *Lithobates catesbeianus*) in natural ponds in SW France where it was introduced about 40 years ago [9]. The method, efficient in detecting frogs even at very low density, can be integrated into the eradication strategy of this invasive species to estimate its distribution in ponds before and after frog removal. Environmental DNA could thus be used for a biodiversity inventory (e.g. introduced Asian carp in North America [10])but also to control the efficiency of eradication actions. In this context, the precise assessment of the species presence requires knowledge of DNA persistence in water.

DNA persistence can be defined as the continuance of DNA after the removal of its source. However, any detection in the field is always imperfect and sampling is a stochastic process [11]. Detection probability depends on the species density and on the ratio between the DNA released by the organism and the DNA degraded by environmental factors.

In this study we estimated the time of DNA detection taking into account aquatic environment conditions and DNA concentrations. Experimentation was performed on two different species: the American bullfrog (*Rana catesbeiana* = *Lithobates catesbeianus*) and the Siberian sturgeon (*Acipenser baerii*).

## Materials and Methods

### Tests conditions and sampling

Two species were used for assessing the persistence of detectable amounts of DNA. Bullfrog tadpoles were studied to validate the possibility of integrating the approach of Ficetola et al. [5] in the species eradication strategy. Due to the risk of invasion and/or pathogen transmission to native populations (e.g. *Batrachochytrium dendrobatidis* [12] and Ranavirus [13]) bullfrog tadpoles cannot be used outdoors, and the experiments were performed in aquariums (as in Ficetola et al. [5]). The siberian sturgeon was used as DNA source species to test field conditions and placed in artificial ponds created about 20 years ago on the University campus, where this species has never been present.

For the bullfrog experiment, 3 different densities of tadpoles were used. One, 5 and 10 tadpoles were reared in 900 mL glass beakers for 5 days and each density was replicated 5 times. A 900 mL glass beaker without tadpoles was used as control. At the fifth day, the tadpoles were removed. At this time and every 24 h during 20 days, 15 mL of water were sampled from each glass beaker. Room temperature was maintained constant throughout the experimental period and the water temperature measured in the glass beakers was 17±1°C.

For the sturgeon experiment, three ponds of dimensions 12 m^2^ and 0.40 m deep were used. In each pond, a sturgeon (20 cm long) was housed for 10 days (from November the 04^th^ to 13^th^ 2009). On the tenth day, the sturgeons were removed and 15 mL of water were sampled from each pond. Water samples were collected every 24 h during 14 days. Water temperature fluctuated from 8 to 11°C during this period.

The duration of each experiment was determined after a preliminary test on the same condition without replication. In both experiments, water samples were added to a solution composed of 1.5 mL of sodium acetate 3 M, and 33 mL absolute ethanol immediately after collection, and then stored at −20°C until the DNA extraction.

### DNA analysis

DNA extraction was adapted from Ficetola et al. [5]: we centrifuged the mixture at 9400 g for 1 h at 6°C to recover DNA and/or the cellular remains. The DNA from the pellet was extracted using QIAmp Blood and Tissue Extraction Kit (Qiagen, GmbH, Hilden, Germany), following manufactures' instructions. DNA extraction was performed in a dedicated room for degraded DNA samples. Control extractions were systematically performed to monitor possible contaminations.

Bullfrog DNA was amplified with primers described in Ficetola et al. [5]. Sturgeon DNA was amplified with primers designed to amplify a 98 bp fragment of the *Acipenser mt*DNA control region (5′ – GACAGTAATTGTAGAGTTTC - 3′and 5′ – CAGTAACAGGCTGATTATG - 3′). *In silico* PCR, performed using the ecoPCR software [14] (http://www.grenoble.prabi.fr/trac/ecoPCR) on the whole GenBank dataset extracted on July 9 2009, showed the suitability of the primer pair. The only 4 species amplified were from the genus *Acipenser*: *A. persicus*, *A. brevirostrum*, *A. gueldenstaedtii* and *A. baerii*, the latter was the only species present in the ponds.

DNA amplifications were carried out in a final volume of 25 µL, using 3 µL of DNA extract as template. The amplification mixture contained 1 U of Ampli*Taq* Gold DNA Polymerase (Applied Biosystems, Foster City, CA), 10 mM Tris-HCl, 50 mm KCl, 2 mM of MgCl2, 0.2 mM of each dNTPs, 0.2 µm of each primer, and 0.005 mg of bovine serum albumin (BSA, Roche Diagnostics, Basel, Switzerland). After 10 min at 95°C (*Taq* activation), the PCR cycles were performed as follows: 55 cycles of 30 s at 95°C, 30 s at 54°C for *A. baerii* and 61°C for *R. catesbeiana* primer pair. The amplification for the sturgeon experiments was repeated 3 times using multi-tube approach [15]. PCR products were visualized using electrophoresis on 2% agarose gel.

For the bullfrog experiment, the DNA detectability was defined as the number of positive samples detected among the 15 samples collected per day (5 replicates and 3 densities). For the Sturgeon experiment, the DNA detectability was defined as the number of positive samples detected among the 9 samples analysed (3 samples collected and 3 PCR per sample).

### Statistical modelling

For the bullfrog experiment, the relationship between the DNA detectability, the time and density of tadpoles was inferred with a generalized linear model using binomial error. For the sturgeon experiment, the relationship between the DNA detectability and time was inferred with a linear mixed model with sites as random effect. In both experiments, a backward selection procedure was used starting with the full model containing all fixed explanatory components. Then, fixed variables were removed step by step. The best fitted model was selected based on AIC [16]. All analyses were done with R (R 2.10) [17].

### Ethics Statement

The research presented has been approved by the Animal Care and Use Committee (permit #CMLECA5553 05/19/05) of the Savoie University at Le Bourget du Lac (France).

## Results

**Figure 1:**
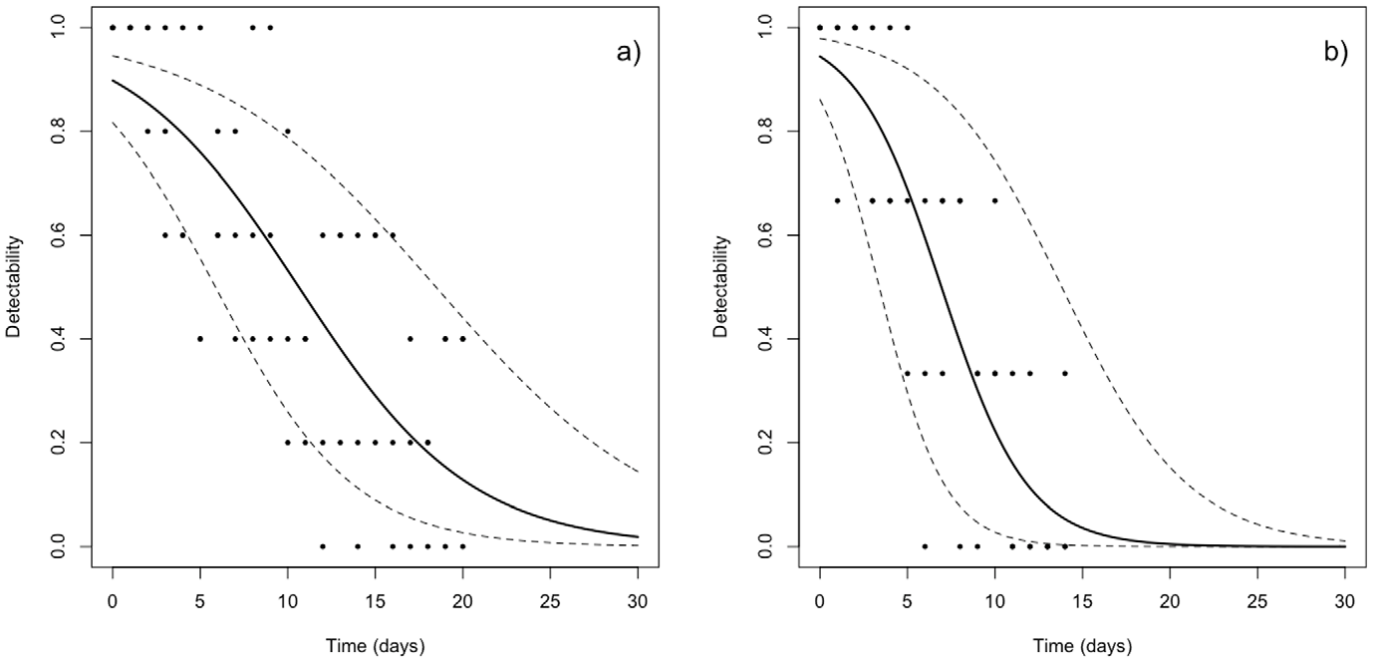
DNA detectability in water according to time. DNA detectability in water in control conditions (a) and in natural conditions (b) according to the time elapsed since the DNA source removal.

In the bullfrog experiment, DNA was detected after tadpole removal at the three densities. DNA detectability was best explained by time and tadpole density factor. DNA detectability (z = −8.032 and p<0.001) was negatively correlated with time. Tadpole density, although significant, showed no trends according to levels of density (no difference between 1 and 5 tadpoles, z = −1.916, p = 0.0553, while DNA detectability was higher with 10 tadpoles compared with 1 tadpole, z = 2.091, p = 0.0365). After the removal of tadpoles, the DNA was detected until day 25 with a detectability superior to 5% (all tadpole densities together; Figure 1a).

In the sturgeon experiment, DNA detectability was negatively correlated with time (z = −6.136 and p<0.001, R^2^ = 0.5). DNA was detected until day 14 with a detectability superior to 5% (Figure 1b). Using 3 replicates per pond, after 17 days there is a probability higher than 95% to not detect short DNA fragments (i.e. the probability that all 3 replicates are negative) or 21 days if a 99% threshold is considered.

## Discussion

Freshwater environments and oceans constitute a great reservoir of extracellular DNA [18]. Its detection in an aquatic environment depends on its release and degradation. The density of individuals influences the dynamics of DNA detectability in water samples, as shown by the bullfrog experiment in this study.

Once released from organisms, extracellular DNA in the environment may persist, adsorbed in organic or inorganic particles. It may also be transformed by competent soil microorganisms, or may be degraded (see [19] for a comprehensive review).

Several factors operate in DNA degradation. Endogenous nucleases, water, UV radiation and the action of bacteria and fungi in the environment contribute to DNA decay [20]. Different studies demonstrated that 300–400 bp fragments could be detected in water up to one week in controlled conditions [21]–[24]. Short DNA fragments are usually very slowly degraded and can be recovered from environmental samples [25]. They are well preserved in dry and cold environments and in the absence of light [20]. For example, the Greenland ancient communities of plants and animals was described using 450 000 year old silty ice samples extracted from the bottom of the Greenland ice cap [26]. In this study, using short fragments, DNA was detectable up to c. a. one month after the removal of its source, for both animal species used. This discrepancy in DNA persistence in for example soil and water and can be due to the action of endogenous nuclease and water that hydrolyses DNA molecules and creates DNA strand breaks by direct cleavage of the DNA phosphodiester backbone or breakage of the sugar backbone after depurination [27]. UV radiation [28] and DNA uptake by micro-organisms, as source of nutriments (carbon, nitrogen and phosphorous) and to repair their own DNA damages [29], contribute also to damage and decrease DNA molecules density. Microorganisms' uptake varies with temperature; and as a consequence, DNA detectability can vary according to the period of the year. In fact low temperature can slow down enzymatic and microbial activity resulting in slower DNA degradation [24].

DNA detection and, as a consequence, DNA persistence estimation, is influenced by sampling and analysis strategy, other than environmental factors. The sampling and the analysis strategy must be extremely rigorous. Before any environmental DNA analysis, the reliability and the robustness of primers must be tested. First, the analysis must be performed *in silico* (e. g. using ecoPCR software [14]) in order to insure primer specificity (e. g. other species were not amplified at the same time as the species of interest) [30]. Once specific primers were found, reliabilty must be tested on very high quality DNA (e.g. extracted from tissus samples), and PCR conditions must be otpimized. Environmental DNA is rare and preacautions similar to those used for ancient DNA analysis must be taken [31]. DNA must be extracted in a dedicated room for rare DNA, mock samples without DNA have to be analysed in parallel, as well as positive samples. PCR cycles have to be increased and high attention must to be taken to avoid contamination. The optimum strategy to enhance the reliability is to increase the number of analysed samples, i.e. more water samples in the field and more genetic replicates (multi-tube approach [15]) in the laboratory. However, all the sampling and analysis strategy must be adapted to the studied environments (e.g. large water bodies, marshes, etc) and species. In running waters, other sampling strategies will be developed, based e.g. on pumping water samples to increase DNA collection.

The dynamics of detectability reflects the persistence of DNA fragments in freshwater ecosystems. In this study we demonstrate that DNA persistence is less than one month. The short time persistence of detectable amounts of DNA opens new perspectives in conservation biology, by allowing access to the presence or absence of species e.g. rare, secretive, potentially invasive, or at low density. This knowledge of DNA persistence will greatly influence planning of biodiversity inventories and biosecurity surveys.
